# Exclusion and Inclusion of Nonwhite Ethnic Minority Groups in 72 North American and European Cardiovascular Cohort Studies

**DOI:** 10.1371/journal.pmed.0030044

**Published:** 2006-01-03

**Authors:** Meghna Ranganathan, Raj Bhopal

**Affiliations:** 1The Robert Wood Johnson Foundation, Princeton, New Jersey, United States of America; **2**Public Health Sciences Section, Division of Community Health Sciences, University of Edinburgh, Edinburgh, United Kingdom; University of BirminghamUnited Kingdom

## Abstract

**Background:**

Cohort studies are recommended for understanding ethnic disparities in cardiovascular disease. Our objective was to review the process for identifying, including, and excluding ethnic minority populations in published cardiovascular cohort studies in Europe and North America.

**Methods and Findings:**

We found the literature using Medline (1966–2005), Embase (1980–2001), Cinahl, Web of Science, and citations from references; consultations with colleagues; Internet searches; and RB's personal files. A total of 72 studies were included, 39 starting after 1975. Decision-making on inclusion and exclusion of racial/ethnic groups, the conceptual basis of race/ethnicity, and methods of classification of racial/ethnic groups were rarely explicit. Few publications provided details on the racial/ethnic composition of the study setting or sample, and 39 gave no description. Several studies were located in small towns or in occupational settings, where ethnic minority populations are underrepresented. Studies on general populations usually had too few participants for analysis by race/ethnicity. Eight studies were explicitly on Caucasians/whites, and two excluded ethnic minority groups from the whole or part of the study on the basis of language or birthplace criteria. Ten studies were designed to compare white and nonwhite populations, while five studies focused on one nonwhite racial/ethnic group; all 15 of these were performed in the US.

**Conclusions:**

There is a shortage of information from cardiovascular cohort studies on racial/ethnic minority populations, although this has recently changed in the US. There is, particularly in Europe, an inequity resulting from a lack of research data in nonwhite populations. Urgent action is now required in Europe to address this disparity.

## Abstract 2

A systematic review reveals a shortage of information on racial and ethnic minority populations. This compromises the relevance of the evidence underlying health policies and guidelines for nonwhite patients.

## Introduction

Cardiovascular disease is the most common cause of death in most industrialised societies and is either the leading or a dominant cause of death for all racial and ethnic groups in the US and the UK. The risk is especially high amongst those originating from the Indian subcontinent—South Asians [1].

Research on ethnic group differences and similarities may potentially help advance understanding of the relationships between risk factors and cardiovascular disease. Cardiovascular cohort studies have been one of the key approaches for achieving such understanding [2,3]. Most such studies started after World War II, when coronary heart disease mortality increased in many western countries [2]. This period coincided with an expansion of migration from developing to industrialised countries, leading to a marked increase in ethnic diversity in Europe and North America in the late 20th century (http://www.migrationinformation.org/GlobalData/countrydata/data.cfm). The inclusion of minority groups in such cohort studies is important not only to compare differences in health status between groups but also to assess risk factor-outcome relationships within such groups. Levy [3] has called for cohort studies to seek answers to ethnic disparities in cardiovascular risks identified in cross-sectional work, while Bhopal and Senior have outlined the problems and potential of ethnicity as an epidemiological variable [4].

The main objective of this review was to identify how the major cardiovascular cohort studies in North America and Europe included or excluded ethnic minority populations. The methods and aims of this review could be extended, but these geographical areas were chosen because cardiovascular cohort studies have been pioneered by groups in these locations [2].

There is no clearly defined line between what is, and what is not, a cardiovascular cohort study, and individual judgment is required to make that determination. For the purposes of this review, cardiovascular cohort studies were defined as prospective studies in defined populations, with a primary aim of studying risk factor-outcome relationships for major diseases such as stroke and coronary heart disease. Studies included are summarised in Table S1 [5–76].

Cohort studies with a multipurpose aim, those focused on other diseases, and those arising from studies originally designed as cross-sectional surveys or trials were generally excluded, as were studies of populations in which the investigators had little or no control over the sample (e.g., volunteers), although they may have yielded some cardiovascular data. A list of the studies that were given careful consideration but excluded, with reasons given, is in Table S2. Our reasoning for focusing on cardiovascular cohort studies, in addition to personal and academic interest, was this: Ethnic variations in cardiovascular disease give a clear rationale for inclusion of ethnic and racial minority groups, which may not be present for other conditions. This review may help health and research policy makers and the research community to judge whether there is equity, by which we mean needs of different populations have been met equally well, and, if not, whether we need new studies.

## Methods

### Search Strategy

The starting point was a preliminary list prepared by RB in 1999. Both authors searched for studies independently between the period April 2000 through September 2005, using a variety of sources and repeated searches. Articles were identified using the electronic databases Medline (1966–2001, repeated in 2003 and 2005), Embase (1980–2001), Web of Science, and Cinahl using the following keywords: “cardiovascular disease” or “atherosclerosis” or “coronary heart disease,” and “cohort studies” or “epidemiological studies” or “prospective studies”. The search was repeated with the words “ethnicity” or “ethnic groups” or “racial groups” added. In Medline the search used free text and MESH terms. This led to more than 150 references in each database. The keywords “ethnicity and cardiovascular disease” used in the Web of Science database yielded more than 300 references. We examined the bibliographies of retrieved articles such as the meta-analysis of prospective observational studies by the Oxford Collaborative Group [2], and searched the Internet using the search engine Google and the Web sites of the British Medical Journal, National Research Register, Medical Research Council, and National Heart, Lung, and Blood Institute. Also, the names of specific cohort studies were keyed into the Internet search engines, e.g., for the Atherosclerosis Risk in Communities (ARIC) Study. Finally, colleagues were consulted, RB's literature files were examined, and referees pointed to additional studies. Grey literature (unpublished reports and abstracts) and editorial correspondence were not included.

The search was limited at the outset to papers in English, as the authors cannot read other languages and most major cohort studies are published in English-language journals. Database filters for English papers only were not applied. Papers with titles in non-English languages were not considered further. Although a count of such papers was not made, our impression is that they were few. Nonetheless, it is unlikely that any important European studies published in languages other than English have been missed. At the Migrant Health in Europe International Conference held in Rotterdam in 2004, RB led a workshop discussing the potential development of a European multiethnic cardiovascular cohort study. Separately, RB presented this paper. The audience of knowledgeable participants were unaware of similar studies in Europe. At the conference many papers on cardiovascular diseases were presented, but none reported such studies. Professor Marc Bruijneels has collected information across Europe, for a proposed European project to compile data by ethnic group but he found no cardiovascular cohort data by ethnic group (personal communication).

Studies were eligible for consideration that were designed to examine prospectively the relationship between risk factors and cardiovascular disease outcomes (coronary heart disease and stroke) in population samples. By population samples we mean natural living populations and exclude studies of people with existing diseases. In view of the nature of this study reflecting how investigators made decisions on who to include and exclude in the sample, studies without a sampling frame based on volunteers were excluded, as investigators would have little decision-making latitude in such circumstances. Some studies were concerned with multiple outcomes, and where publications showed an emphasis on cardiovascular diseases these were included, e.g., the Nurses Health Study [63]. Cohort studies where the sample was defined retrospectively based on records (investigators have limited choices over sampling and on ethnic coding in such studies), trials, cross-sectional studies, case control studies, and studies based solely on routine statistics were excluded. Inclusion decisions required a degree of judgment and flexibility because, as stated above, there is no firm definition for a cardiovascular cohort study. Furthermore, investigators themselves conducted and analysed their studies flexibly, and assessing the study design was not always easy, e.g., with follow-up of studies that were originally cross-sectional. We also followed advice from referees, e.g., The Women's Health Initiative observational study was included, although a major component of this study is a trial. Multiple Risk Factor Intervention Trial (MRFIT) was designed as a trial, although it contained an observational component (with volunteer samples), and was excluded. Studies designed to study cancer, e.g., the European Prospective Investigation of Cancer (EPIC) [77] and the Multi-Ethnic Los Angeles Cohort Study, were excluded (Table S2), although such studies may shed light on cardiovascular diseases.

In total, 72 cardiovascular cohort studies in the US/North America and Europe were included. This review focused on papers describing the design and rationale of the study. For example, the Framingham Study has hundreds of secondary papers, but a few discussing methods were identified. This approach is justified on both scientific and pragmatic grounds. Scientifically, inclusion/exclusion of particular populations is a design issue that is handled at the planning stage. Pragmatically, it would have been inefficient to examine multiple papers for information that ought to be provided in the baseline paper. Only one paper per study is cited here, although sometimes several were examined. Studies based on combining existing cohorts, e.g. the Sleep, Heart and Health Study (Table S2), are not included here, but the original studies are when appropriate.

### Research Questions and Data Extraction

The research questions that guided data collection from the studies are listed in Table 1. Information extracted from publications was directly entered into Table S1. Both authors independently examined all the papers with virtually complete agreement. The few disagreements were resolved by conferring.

**Table 1:**
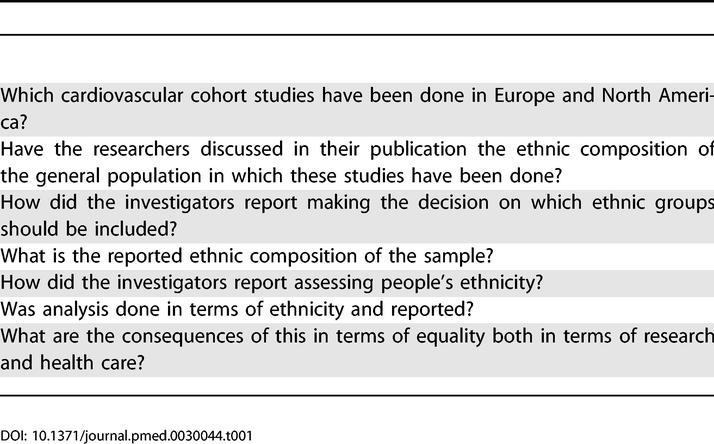
Research Questions

### Terminology and Concepts of Race and Ethnicity

Wherever possible and appropriate, the terminology used for ethnic group classification has been quoted directly from the paper, even when this is not in agreement with currently accepted terminology and is potentially offensive. Similarly, we have accepted the concepts of race and ethnicity provided by authors, but for reasons discussed by Senior and Bhopal we have tended to use the word ethnicity rather than race, and we apply the concepts as discussed in their paper [4].

Our use of the term nonwhite reflects our focus on populations that do not have European ancestral origins (described, using current conventions, as white), and would not describe themselves, or be perceived as, white. This focus reflects long-standing, widespread concern about inequities in health and health care that are particular to such populations.

## Results

### Overview

The main aim of each study, with slight variations, was to determine the incidence of coronary heart disease and/or stroke and study risk factor-outcome relationships.

Table 2 summarises and Table S1 lists the 72 studies included [5–76]. The studies started between 1946 and 2000, with 39 starting after 1975, by which time ethnic minority populations were becoming well established in Western Europe (http://www.migrationinformation.org/GlobalData/countrydata/data.cfm, and knowledge of ethnic variations in cardiovascular disease was appearing in Europe. Studies numbered 41 in Europe, 31 in North America, and one (the Seven Countries study) in both. Ten studies were designed to compare white and nonwhite populations, while five studies focused on one nonwhite racial/ethnic group; all 15 of these were conducted in the US.

**Table 2:**
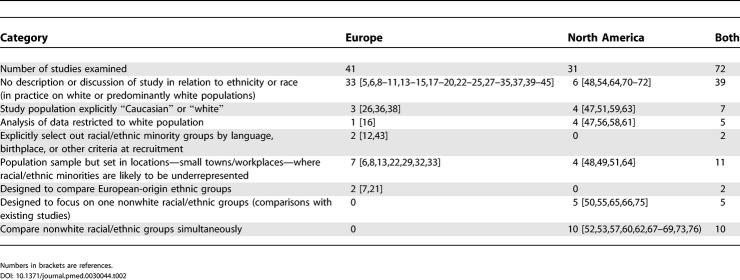
Summary of Key Findings in Table S1: Inclusion and Exclusion of Racial/Ethnic Minorities in Cardiovascular Cohort Studies

Studies seldom provided details on the racial or ethnic composition of the study setting or sample, and when they did the details were minimal; 39 gave no description at all. Several studies were located in small towns or in occupational settings, whereas minority populations tend to live in cities and work in a restricted range of workplaces. The investigators in some studies saw the population homogeneity of such locations as valuable. Studies that were based on general populations usually had too few participants for analysis by race or ethnicity. The process by which decisions on inclusion and exclusion of racial/ethnic groups were rarely made explicit. Eight studies explicitly stated they were on Caucasians or whites, and two excluded ethnic minority groups using language and birthplace criteria. One study [61] excluded the nonwhite population (6,236 people) from the incidence component of the research because of small numbers and low response rates to mailed questionnaires. There were other examples of studies including ethnic minority groups in the baseline phase of the study but reporting cohort analyses in the white population. There were major differences in the extent of inclusion of minority groups between Europe and the US.

### Europe

None of the studies done in Europe mentioned studying nonwhite racial or ethnic variations in their aims. The ethnic composition of the source population for the sample was not described or discussed, but sometimes the text showed awareness of the issue of ethnic heterogeneity; e.g., the Paris Prospective Study [12] was explicitly of native-born French men, while the Second Manifestations of ARTerial disease (SMART) study [43] was of Dutch speakers. Two studies intended to examine European origin ethnic groups. The Yugoslavia Cardiovascular Disease Study [7] contrasted Muslim and Roman Catholic populations, but there was little detail on their characteristics. The Cardiovascular Disease in Norwegian Counties Study [21] collected information by ethnic group but provided no analysis on this variable. Few authors specified the ethnic composition of their sample, and when they did it was usually with the label Caucasian or white with no or sparse detail on the assignment of ethnic/racial group.

### North America

Most North American studies gave some attention to the issue of ethnicity and race, usually in relation to the sample rather than the racial/ethnic composition of the setting of the study. The assignment of racial/ethnic group, and its validity, were not made explicit. Five studies focused on one nonwhite group: the Meharry Cohort Study [50] focused on African-American students; the Jackson Heart Study [75] focused on a black population; the Gila River Indians Community Study [65] studied adult American Indians (Pimas), as did the Strong Heart Study (12 American Indian tribes in Arizona, Oklahoma and North and South Dakota) [66]; and the NI-HON-SAN project [55] studied adults of Japanese origins, comparing American and Japanese locations.

Ten studies compared one or more ethnic groups simultaneously: the Evans County Study [52], the ARIC study [69], and the Charleston Heart Study [53] compared black and white adults; the CARDIA Study [67] compared black and white students; the Bogalusa Heart Study [60] compared black and white school children; the San Antonio Heart Study compared cardiovascular risk factors in Mexican Americans and Anglos [68]; the National Health and Nutrition Examination Survey (NHANES) [57], the Multi-Ethnic Prospective Cohort [62], the Women's Health Initiative [73], and the Multi-Ethnic Study of Atherosclerosis [76], were multiethnic studies.

## Discussion

The study of ethnic variations in disease is long established, with a research base founded in the 19th century and strengthening in the 20th century, particularly in North America. The cardiovascular research community has been aware for some decades of important variations in the frequency, causes and consequences of cardiovascular diseases in non-European origin ethnic minority populations. Well-publicised government reports and academic papers discussed these issues in the mid-1970s and 1980s, e.g., heart attacks in East London were shown to be high in Asians (mainly from the Indian subcontinent) and low in Caribbeans [78]. Mortality rates may vary threefold or more between minority ethnic groups; e.g., in a comparison of Chinese and South Asian populations living in the UK [1], the variations were much larger than those among white minority populations, e.g., the Irish-born people living in England and Wales compared to the whole population there [79]. There is no published major cohort study focusing on ethnic group variations in Europe, but a growing information base is developing in North America. This observation is important for both policy and practice. For example, risk prediction models have been developed from data on white European origin populations, and their unreliability in relation to racial/ethnic minority groups is recognised [80, 81]. Within the cardiovascular field there is also concern about possible racial disparities in health care and outcomes [82,83]. Ethnic minority groups in the US and in Europe are “at-risk” of differential treatment, particularly for surgical therapies, and several explanations, including institutional discrimination, are being pursued [83]. The decisions of most individual investigators undertaking cohort studies to concentrate on white European origin populations may have been scientifically sound and well meant, but collectively, especially in Europe but also for some ethnic groups in the US, it may have resulted in a lack of attention to the needs of nonwhite populations.

### Limitations of This Study

Since this review was limited to papers published in English, there was the potential to miss relevant, large-scale cardiovascular cohort studies in Europe and North America published in other languages. The reference lists of papers examined did not, however, cite them, and consultations around Europe did not identify them (see Methods). Bias from such omissions, if any, is unlikely to alter the conclusions of this paper. One or a few papers from each study, usually those giving adequate detail on the rationale and design of the study (sample, participants, methods used, etc.), were studied. Secondary papers may mention ethnic groups, as in the Whitehall Study, where cross-sectional analyses were done [84]. This said, the papers we studied clarified the primary intentions and design of each study. It is axiomatic that unless the race/ethnicity component is considered at the design stage, and the ethnic group of participants is identified, useful data on this issue are unlikely to accrue later. Our search strategy, which excluded manual searching of journals, may have missed some studies, as was also acknowledged by The Prospective Studies Collaboration [2]. It would be an inefficient, and possibly futile, exercise to catalogue every study, especially as several cohorts have led to hundreds of papers. However, to our knowledge, this is the most complete list of cardiovascular cohort studies available. We have excluded small-scale studies that were combined to create a cohort, e.g., the Italian RIFLE project consisting of 45 “cohorts” and a total sample of 32,726. There is no mention of race or ethnicity in the paper [85]. It is improbable that such small individual studies included the ethnic dimension. The studies included meet the highest standards, as indicated by publication in journals indexed by electronic databases. There are many other cohort studies that are multipurpose or focus on noncardiovascular diseases. In theory these could potentially yield data on ethnic variations in cardiovascular diseases. Our assessment of such studies suggests that they give no more detail on the racial/ethnic issues than those we have examined (Table S2). For example, the EPIC-Norfolk study did not discuss ethnicity [77].

Unpublished (grey) literature has not been included in this review, with the exception of the Jackson Study [75], which links to the published ARIC study and is fully described on a website. This exception was made because of its obvious importance. We are aware of some cross-sectional studies (generally small) that are designed with linkage to mortality follow-up, e.g., the Southall Study [86] and the Newcastle Heart Project [87], that will publish risk factor-outcome data in due course. These were, however, designed with the power for cross-sectional and not cohort analyses. A cohort study of Indian Asians in West London is ongoing (Jaspal Kooner, personal communication), but this study will not address the needs of other minority ethnic groups in the UK. There are also many studies designed as trials that have long-term follow-up and provide opportunities for cohort type analysis, e.g., MRFIT [88]. Analysis of inclusion and exclusion of ethnic minority populations in trials and other study designs was beyond the scope of this study, although it may be that the findings will be similar. Further research on cardiovascular trials, cross-sectional studies, and case-control studies might be illuminating. These limitations do not, however, alter our main conclusions.

### Interpretation of Main Results

The review answered the research questions (Table 1). There are many cardiovascular cohort studies, indicating their perceived and actual importance. The ethnic composition of the population where the studies were based, and the process of inclusion/exclusion of ethnic minority groups, was not a point of emphasis in publications. Many studies gave little or no data on the ethnic composition of the sample, or the description was limited and based on ethnic group labels. With the exemplary exception of the San Antonio study, which developed an algorithm based on a range of data, studies did not provide details of the processes for ethnic coding. Cohort-based analysis by ethnic group is available in the US for a number of ethnic groups, but not in Europe.

Although the sample size may be too small to produce analysis by ethnic group, inclusion of minority populations is still important. Such cohorts are population-based and should be generalisable to populations similar to that from which the sample has been drawn; without information on ethnic composition, generalisability becomes more difficult. Such studies can also provide a foundation for larger studies focusing on minority populations, and potentially could lead to analysis by ethnic group after pooling of data.

Analysis of data by ethnic group requires adequately powered studies, and these will be large-scale, expensive, and challenging. Such studies will be funded only when there is agreement on the need for them. There are more than 30 European cohort studies, many started within the last 20 years when ethnic variations were already described, yet collectively or singly they are unable to provide analysis by ethnic group. This paper contributes to the needs assessment.

Many cohort studies have focused on white populations despite being set in multiracial/ethnic nations and regions (http://www.migrationinformation.org/GlobalData/countrydata/data.cfm). This is especially so in European studies, e.g., those set in major cities such as London, Amsterdam, and Paris. This observation applies to studies started after the mid-1970s when understanding about the needs of minority groups was substantial, many cohort studies on white populations were in place, and knowledge about causes and control of cardiovascular disease in white populations was already advanced. The studies that were designed to study racial/ethnic minority groups were all in the US, and were started more recently, in response to an increasing recognition of the needs of ethnic minorities.

Some studies were openly exclusive, e.g., including only the native born or native language speakers, or simply being confined to whites/Caucasians. Some studies used members of occupational groups as study participants. This can lead to exclusion of minority populations, perhaps unwittingly, in that unemployment is usually higher in ethnic minority populations, some of which have comparatively high levels of self-employment and employment in small workplaces and are less likely to be a substantial proportion of the work force of large employers [89]. While recruiting randomly is, arguably, fair, it rarely permits analyses by ethnic group, because the resultant sample size is too small, except for European-origin populations. This can lead to incidence data analyses in white populations and more limited analysis, e.g., cross-sectional in ethnic minority groups [84]. By choosing small towns or rural areas, as their base, as in the Framingham, Tecumseh, Seven Countries, Caerphilly, and British Regional Heart Studies, investigators gain population stability and homogeneity but miss multiethnic populations living predominantly in inner city areas, where the cardiovascular disease prevention challenge is greatest. In these circumstances there is a case for more purposive sampling, including weighting the sample to augment the number of ethnic minority participants. Studies that started after 1975 and which, in retrospect, might have been designed in this way include Whitehall 2, Rotterdam Elderly Study, British Women's Heart and Health Study, Nurses Health Study, Iowa Women's Health Study, and the Health Professionals Heart Study. With the exception of black/white comparisons, the opportunity for multiethnic comparison has not been fully exploited, although several recent studies in the US promise a truly multiethnic approach.

Explanations for the findings here include scientific pragmatism, shortage of resources, potential difficulties in accessing populations and in gaining informed consent, insufficient expertise and experience, lack of interest, a resistance to dividing populations by ethnic or racial status (particularly in some countries of mainland Europe), and the possibility of indirect or direct discrimination. In many ways the issues highlighted echo those applying to women until recently. These explanations require further analysis and research.

Data are vital to assess the needs of ethnic minority groups; to implement, evaluate, and adjust the necessary health policies; and to provide excellent clinical care based on valid risk prediction models. The Race Relations (Amendment) Act 2000 in the UK [90] and laws in Europe will mandate a change of strategy for all public sector organisations, including those that commission or fund research, such as the Medical Research Council. In the US, the NIH Strategic Research Plan (2002–2006) promises to spearhead further change, building on previous NIH policies [91]. This paper indicates that researchers in the US have responded to NIH policies promoting the inclusion of ethnic minority populations in research. Studies exploring ethnic variations may lead to insights that are generalisable to the whole population, in terms of both disease causation and the effectiveness of interventions and healthcare systems. Inclusion of ethnic minorities groups in research, therefore, is likely to benefit the whole population.

A Lancet commentary has called for cohort studies in such groups [3]. The traditional approach, whereby researchers', peer reviewers', and funding bodies' interests drive the research agenda, needs to be balanced by a strategic needs-based approach if the inequity described in this paper is to be addressed. The planned Biobank UK study of a cohort of 500,000 people in the UK offers an opportunity to redress the gap in the UK—but only if it achieves its stated goals of recruiting ethnic minority groups with due emphasis on population heterogeneity, attention to cross-cultural comparability of data, and high retention of ethnic minority populations in the cohort (http://www.ukbiobank.ac.uk/). This paper raises broader questions that merit debate on how the research community responds to the increasing ethnic diversity of populations internationally.

## Supporting Information

Table S1 Cardiovascular Cohort Studies Included by Area of Study (Europe and North America) and in Chronological Order of Fieldwork Found at 10.1371/journal.pmed.0030044.st001 (139 KB DOC).

Table S2 List of Studies Excluded from the Analysis Found at 10.1371/journal.pmed.0030044.st002 (41 KB DOC).

**Patient Summary**

**Background**

As a result of migration, mostly for economic reasons, populations in many countries around the world are becoming increasingly diverse. In many cases, ethnic minorities differ in their socio-economic circumstances, culture, lifestyle, and genetic make-up from the majority population (and there are, of course, also differences within the majority group and within minority groups). These differences in risk factors (such as diet and smoking) can influence a person's susceptibility to disease. For some diseases, such as heart disease, it is well known that particular ethnic groups are at higher risk than others. It is not always clear, though, whether this high risk is due to socio-economic circumstances, culture, genes, lifestyle, or a combination of these factors.

**Why Was This Study Done?**

Cardiovascular disease, which can cause heart attacks and strokes, is the most common cause of death in the US and most European countries. Rates of cardiovascular diseases vary substantially between different countries, and also between different ethnic groups in ethnically diverse countries such as the UK and the US. Researchers and health policy makers need to understand more about the variations in cardiovascular disease. To ensure the best possible health care for the entire population, they need to know how exactly the risks differ between different ethnic groups and what causes those differences. One key research tool to address these questions are so-called cohort studies (a cohort refers to a specific group of people that are studied over time). Cohort studies are “forward looking”—they typically enroll a large number of healthy participants who are then followed over a number or years to study long-term health outcomes. Over the past decades, a number of cohort studies have focused on cardiovascular disease. In this study, researchers wanted to find out whether these cohort studies included or excluded ethnic minority groups.

**What Did the Researchers Do and Find?**

They searched the medical literature for cohort studies on cardiovascular disease. They found 72 that met their criteria and analyzed them in detail. The researchers discovered that most of the cohort studies did not provide detailed information on the ethnic composition of the broader populations from which the participants were recruited. Most of them also did not state whether minority groups were included in or excluded from the study. Additionally, the researchers found that many of the studies did not give details on the ethnic composition of the participants themselves, or on how the participants' ethnicity was determined. Studies with participants that were representative of diverse populations usually were not large enough to answer the kinds of questions necessary to determine differences between different ethnic groups. However, ten of the studies were designed specifically to compare white and nonwhite participants, and five studies focused on nonwhite minority groups specifically. All 15 of those studies were done in the US.

**What Does This Mean?**

Despite the knowledge that ethnicity matters in cardiovascular disease, most cohort studies have not been designed to further explore this connection. The situation in the US seems to be changing, with a number of recent studies designed to add data on cardiovascular disease risks and causes among minority populations. No such studies have yet been reported in Europe. Research strategies in Europe should be adjusted to meet this need.

**Where Can I Find More Information Online?**

The following Web sites provide information on minority participation in health research.

Office of Minority Health Research at the US National Heart, Lung, and Blood Institute:

http://www.nhlbi.nih.gov/about/omha/

Center of Excellence in Minority Health and Health Disparities at Harvard Medical School:

http://www.mfdp.med.harvard.edu/coe/

The Migration Information Source provides data on the composition of the populations for many countries:

http://www.migrationinformation.org/

Department of Health, England:

http://www.dh.gov.uk/PolicyAndGuidance/EqualityAndHumanRights/fs/en/

South Asian Health Foundation:

http://www.sahf.org.uk/events.html

## Abbreviations

ARIC: Atherosclerosis Risk in Communities
MRFIT: Multiple Risk Factor Intervention Trial
