# Supplementary material for: Exclusion and Inclusion of Nonwhite Ethnic Minority Groups in 72 North American and European Cardiovascular Cohort Studies
Source: PLoS Med. 2006 Jan 3;3(3):e44. doi: 10.1371/journal.pmed.0030044 (PMC1324792; doi:10.1371/journal.pmed.0030044)
Supplement: Supplementary file 1 [file pmed.0030044.st001.doc]

**Table S1. (for probable publication as appendix)**

**Cardiovascular cohort studies included by area of study (Europe**

and North America) and in chronological order of fieldwork

| ***Name of cohort study***  ***(reference)*** | ***Start of fieldwork*** | *Place* | ***Sample size*** | ***Authors’ comments on concepts/classification and inclusion relating to race or ethnicity*** |
| --- | --- | --- | --- | --- |
| **Europe** |  |  |  |  |
| Study on London Busmen^5^ | 1956 | London | 687 | No description or discussion. |
| Seven Countries Study^6^ | 1957 | Finland, Greece, Italy, Japan, The Netherlands, USA, Yugoslavia | 12,293 | No within-country consideration of ethnicity and race. |
| Yugoslavia Cardiovascular Disease Study^7^ | 1964 | Tugla, Bosnia and Remetinec, Zagreb | 11,121 | Deliberate choice of contrasting Muslim and Roman Catholic populations, with differing cultures – no further detail. |
| Belgian Bank Study^8^ | 1964 | Belgium | 1,415 | No description or discussion |
|  |  |  |  |  |
| The Social Insurance Institution’s Coronary Heart Disease Study^9^ | 1966 | Four regions of Finland | 10,962 | No description or discussion |
|  |  |  |  |  |
| Reykjavik study^10^ | 1967 | Reykjavik and surroundings, Iceland the long (phased recruitment) | 31,000 | No description or discussion |
| Whitehall-1 Study^11^ | 1967 | Whitehall, London, UK | 18,403 | No description or discussion. |
| Paris Prospective  Study^12^ | 1967 | Paris, France | 7,746 | Choice of sample – native born French men – excludes some groups. |
| Gothenburg Study^13^ | 1968 | Gothenburg, Sweden | 1,462 | No description or discussion. |
| Manresa Study^14^ | 1968 | Manresa, Catalonia, Spain | 1,059 | No description or discussion |

| ***Name of cohort study***  ***(reference)*** | ***Start of fieldwork*** | Place | ***Sample size*** | ***Authors’ comments on concepts/classification and inclusion relating to race or ethnicity*** |
| --- | --- | --- | --- | --- |
| Copenhagen Male Study^15^ | 1970 | Copenhagen,  Denmark | 5,249 | No description or discussion. |
| Northwick Park Heart Study^16^ | 1972 | North-West London | 3,500 | Occupational setting with white, black and ’other’ groups. Prospective analysis only on white population. |
| Oslo Study^17^ | 1972 | Oslo, Norway | 17,965 | No description or discussion. |
| Midspan Study^18^ | 1972 | Paisley and Renfrew, Scotland | 15,411 | No description or discussion. |
| N Karelia/ Kuopio Study^19^ | 1972 | N Karelia/ Kuopio, Finland | 28,600 | No description or discussion. |
| Tromso Study^20^ | 1972 | Tromso, Norway | 27,721 | No description or discussion |
| Cardiovascular Disease study in Norwegian Counties^21^ | 1974 | Norway  (counties of Finnmark, Siegn og Fjordane, Oppland) | 66,200 | In Finnmark information requested on ethnic origin; material produced in Lapp; place of birth recorded. No analysis by ethnic group. |
| The British United Provident Association (BUPA) Study^22^ | 1975 | London, UK | 21,515 | No description or discussion |
| Pilsen Longitudinal Study ^23^ | 1976 | Pilsen, Czech Republic | 3,758 | No description or discussion. Occupational sample. |
| Copenhagen City Heart Study^24^ | 1976 | Copenhagen, Denmark | 12,149 | No description or discussion |
| Danish Glostrup Study^25^ | 1976 | Copenhagen, Denmark | 10,092 | No description or discussion |
| Nijmegan cohort study^26^ | 1977 | Nijmegan, East Netherlands | 7,092 | Sample of Caucasians; no description of exclusion of non-caucasians. |
| The Amsterdam Growth and Health Study^27^ | 1977 | Amsterdam,  The Netherlands | 182 | No description or discussion |

| ***Name of cohort study***  ***(reference)*** | ***Start of fieldwork*** | ***Place*** | ***Sample size*** | ***Authors’ comments on concepts/classification and inclusion relating to race or ethnicity*** |
| --- | --- | --- | --- | --- |
| British Regional Heart Study (BRHS)^28^ | 1978 | 24 towns in England,Wales and Scotland | 7,727 | No description or discussion. |
| Munster Heart Study^29^ | 1979 | North-West Germany | 16,288 men and 7,328 women | No description or discussion. Occupational cohort of 52 companies and local government authorities. |
| The Belgian Interuniversity Research on Nutrition and Health (BIRNH) Study^30^ | 1980 | 43 districts of Belgium | 11,302 | No description or discussion |
| Malmo ‘men born in 1914’ study^31^ | 1982 | Malmo, Sweden | 484 | No description or discussion. |
| Caerphilly and Speedwell Collaborative Studies^32^ | 1983 | Caerphilly, Wales  Speedwell, Bristol | 2,300 in each place | No description or discussion. |
| Gubbio Population Study^33^ | 1983 | Gubbio, Italy | 2,469 | No description or discussion. Based on population of one small town. |
| Kuopio Ischemic Heart Disease Risk Study^34^ | 1984 | Eastern Finland | 11,130 | No description or discussion. |
| Whitehall –2 Study^35^ | 1985 | London, UK | 10,314 | No description or discussion; cross-sectional data were subsequently published on South Asians and African-Caribbeans. |
| CORALI Study^36^ | 1987 | France  (towns of Lille, Lyon, Paris, Toulouse) | 484 | Caucasian sample; exclusion of non-Caucasians not discussed. |
| Scottish Heart Health Study^37^ | 1984 | Scotland | 10,359 | No description or discussion. |
| Hoorn Study ^38^ | 1989 | Hoorn, Holland | 2,484 | White population; exclusion processes not described or discussed. |

| ***Name of cohort study***  ***(reference)*** | ***Start of fieldwork*** | ***Place*** | ***Sample size*** | ***Authors’ comments on concepts/classification and inclusion relating to race or ethnicity*** |
| --- | --- | --- | --- | --- |
| Rotterdam Elderly Study^39^ | 1990 | Oomoord, Rotterdam, The Netherlands | 7,983 | No description or discussion. |
| Bruneck Study^40^ | 1990 | Bruneck, Italy | 474 | No description or discussion. |
| Edinburgh Artery Study^41^ | 1991 | Edinburgh, UK | 1,500 | No description or discussion. |
| Prime Study^42^ | 1991 | France  (Lille,Strasbourg and Toulouse)  and Northern Ireland  (Belfast) | 10,592 | No description or discussion. |
| SMART (Second Manifestations of ARTerial disease) Study^43^ | 1996 | Utrecht, The Netherlands | 778 | Dutch speakers only. |
| Goettingen Risk, Incidence and Prevalence Study (GRIPS)^44^ | 1996 | Goettingen, Germany | 5,790 | No description or discussion. |
| British Women’s Heart and Health Study^45^ | 1999 | 24 towns in England, Wales and Scotland | 2,341 | No description or discussion. |
| **North America** |  |  |  |  |
| Manitoba Follow-up Study^46^ | 1946 | Manitoba, Canada | 3,983 | Mixed population of pilots from several countries – no analysis by birthplace. |
| The Johns Hopkins Precursor Study^47^ | 1947 | Baltimore, USA | 1337 | Students at Johns Hopkins Medical School. Key analysis reported on 1,017 white men. |
| Framingham Study^48^ | 1948 | Framingham, Massachusetts,  USA | 5,209 | No description or discussion. |
| Chicago Western Electric Company Study^49^ | 1957 | Chicago  (Hawthorne works of W. Electric company) | 1,989 | Occupational sample of predominantly Polish and Bohemian ancestry – no further discussion. |

| ***Name of cohort study***  ***(reference)*** | ***Start of fieldwork*** | ***Place*** | ***Sample size*** | ***Authors’ comments on concepts/classification and inclusion relating to race or ethnicity*** |
| --- | --- | --- | --- | --- |
| Meharry Cohort Study^50^ | 1958 | USA | 435 | African-American medical students of Meharry Medical College from 1958-1965. |
| Tecumseh Study^51^ | 1959 | Tecumseh, Michigan, USA | 8,641 | Set in town of almost entirely Caucasian ancestry – no further details. |
| Evans County Cardiovascular Disease Study^52^ | 1960 | Claxton, Evans County  Bulloch County, (USA) | 3,390 | Compares white and negro populations – no discussion of concepts and classification. |
| Charleston Heart Study^53^ | 1960 | Charleston, South Carolina, USA | 2,275 | Compares black and white people – no discussion of concepts and classification. |
| College Alumni Health Study^54^ | 1962 | Philadelphia, PA,  USA | 1564 | Female University of Pennsylvania alumni- no discussion of race or ethnicity |
| Honolulu Heart Program  (NI-HON-SAN Study)^55^ | 1965 | Oahu, Hawaii,  USA | 28,193 | Population of Japanese ancestry – no discussion of concepts and classification, but there is a description of the migratory pattern. |
| Chicago Heart Association Detection Project in Industry^56^ | 1967 | Chicago, USA | 39,572 | Enrols a mixed ethnic/racial sample but no analysis by ethnic/racial group. |
| NHANES-1  (National Health and Nutrition Examination Survey) ^57^ | 1971 | USA | 13,643 | Main comparisons are for white and black groups – some other comparisons possible. |
| Muscatine Study^58^ | 1971 | Muscatine, Iowa,  USA | 4,829 | Provides ethnic composition of population (4% non-white), but no analysis. |

| ***Name of cohort study***  ***(reference)*** | ***Start of fieldwork*** | ***Place*** | ***Sample size*** | ***Authors’ comments on concepts/classification and inclusion relating to race or ethnicity*** |
| --- | --- | --- | --- | --- |
| Rancho Bernado Study^59^ | 1972 | Rancho Bernado, California, USA | 3,456 | Mentions that the sample setting is predominantly white and results may not be generalisable to other groups. |
| Bogalusa Heart Study^60^ | 1973 | Louisiana, USA  (Includes Bogalusa) | 8,000 | Compares black and white schoolchildren – no discussion of concepts or classification. |
| Adventist Health Study^61^ | 1974 | California, USA | 34, 198 | Non-Hispanic white Seventh-day Adventists. 6,236 non-white others excluded from incidence element of study. |
| Multiethnic Prospective Cohort^62^ | 1975 | Hawaii, USA | 40,000 | All ethnic groups in Hawaii including Caucasian, Chinese, Filipino, Japanese and native Hawaiian ethnicity. |
| Nurses Health Study^63^ | 1976 | USA | 121,700 | Female registered nurses from 11 US states. On a subsample of 249 nurses, estimated that 98% of the sample is white. |
| Harvard Alumni Health Study^64^ | 1977 | Harvard, Cambridge, USA | 11,130 | No description or discussion. |
| Gila River Indian Community Study^65^ | 1979 | Arizona,  USA | 3,050 | Studies Pima Indians. |
| Strong Heart Study^66^ | 1984 | Central Arizona, S. W. Oklahoma and the Aberdeen Area of North and South Dakota. | 4,500 | Studies several American Indian tribes. |

| ***Name of cohort study***  ***(reference)*** | ***Start of fieldwork*** | ***Place*** | ***Sample size*** | ***Authors’ comments on concepts/classification and inclusion relating to race or ethnicity*** |
| --- | --- | --- | --- | --- |
| CARDIA  (Coronary Artery Risk Development in Young Adults).^67^ | 1984 | Birmingham,  Alabama  Chicago, Illinois  Minneapolis,  Minnesota  Oakland, California | 5,116 | Black and white students. Other racial groups sometimes examined but excluded from analysis – no details on concepts and classification. |
| The San Antonio Heart Study^68^ | 1984, 1979 | San Antonio, Texas | 2,217 | 1,288 Mexicans and 929 Anglos (the local term for ‘white’). Ethnicity was assigned using an algorithm including parental surnames and birthplace, grandparental ethnicity and participant’s preferred identity. |
| Atherosclerosis in the community (ARIC)^69^ | 1986 | N. Carolina, Mississippi, Minnesota, Maryland (USA) | 16,000 | Compares white and black groups – no details on concepts or classifications. |
| Iowa Women’s Health Study^70^ | 1986 | Iowa, USA | 41,386 | No description or discussion. |
| Health Professionals Heart Study^71^ | 1986 | USA | 43,757 | No description or discussion |
| The Cardiovascular Health Study (CHS)^72^ | 1989 | USA (N. Carolina,  California, Maryland,  Pennsylvania) | 5,201 | No description or discussion in baseline paper (noting that 95 percent of the original participants were white, 687 people from minority ethnic groups were added in 1992-1993). |

| ***Name of cohort study***  ***(reference)*** | ***Start of fieldwork*** | ***Place*** | ***Sample size*** | ***Authors’ comments on concepts/classification and inclusion relating to race or ethnicity*** |
| --- | --- | --- | --- | --- |
| Women’s Health Initiative^73^ | 1992 | National, USA – 40 centres. | 100,000 in observational study. | 20% target for racial/ethnic minority groups, with weighting in favour of their inclusion. No details on concepts or classifications, but there is a statement that African-Americans and Hispanic Americans will be actively recruited. Black/white comparisons have been reported. |
| The Los Angeles Atherosclerosis Study^74^ | 1998 | Employees of a utility company | 573 | Oversampling of Hispanics. |
| Jackson Heart Study^75^ | 2000 | Jackson, Mississippi,  USA | 6,500 | African American. |
| Multi-ethnic study of Atherosclerosis^76^ | 2000 | Six USA centres - New York, Baltimore, Chicago, Minneapolis, Los Angeles, Winston-Salem. | 6,500 | 40% white, 30% African-American, 20% Hispanic, 10% Asian (mostly Chinese). |
