# Supplementary material for: Exclusion and Inclusion of Nonwhite Ethnic Minority Groups in 72 North American and European Cardiovascular Cohort Studies
Source: PLoS Med. 2006 Jan 3;3(3):e44. doi: 10.1371/journal.pmed.0030044 (PMC1324792; doi:10.1371/journal.pmed.0030044)
Supplement: Supplementary file 2 [file pmed.0030044.st002.doc]

| **Table S2.** |  |  |
| --- | --- | --- |
| **Study** | **Reference** | **Reasons for Exclusion** |
| Aberdeen cohort of hypertension in pregnancy and outcomes | BMJ 2003:326: 845-9 | Specific, not general population, sample and retrospective cohort. |
| European Prospective Investigation into  Cancer and Nutrition (EPIC) | Br J Cancer 1999; 80:95-103 | The focus of the cohort is cancer, even though cardiovascular outcomes are studied. |
| Finnish 10-Town Study | BMJ 2004; 328: 555-563 | Cardiovascular mortality was one of several outcomes studied for aims that were not those of a cardiovascular cohort study. |
| Lipid Research Clinics Program Prevalence Study;  and Follow-up Study | Circulation 1979; 60:427-439  Circulation 1987; 75:1102-1109 | The study was designed as a cross-sectional one. |
| Minnesota Heart Health Program | J Chronic Diseases 1986;39:775-788 | The study is an evaluation of a community based intervention and is cross-sectional. Small cohort studies are subsidiary to this aim. |
| MONICA studies e.g. MONICA Augsburg | Circulation 2004; 109:1349-1353 | The MONICA studies were designed as cross-sectional studies. |
| Multiethnic Prospective Cohort Study | Epidemiology 1998; 9: 654 - 61 | Focus on cancer outcomes not cardiovascular disease. |
| Multiple Risk Factor Intervention Trial (MRFIT) | Preventive medicine 1981; 10: 402-425 | The screening study, which led to the MRFIT cohort, was done primarily to select people for the CHD prevention trial. The screening was not in defined populations. |
| National Health Interview survey | Am J Epidemiology 1999;149:41-46 | Multipurpose cross-sectional surveys linked to mortality outcomes. Not a cardiovascular cohort study. |
| National Heart, Lung, and Blood Institute (NHLBI) Family Heart Study | Am J Epidemiology 1996; 143:1219-28 | The cohort is recruited from existing (parent) studies. |
| Normative Ageing Study | Ann Epidemiology 1990; 1(1): 33-48. | Not designed as a cardiovascular cohort and it does not produce disease outcomes data. |
| Northern Californian Kaiser Permanente Study | Int. J. Epidemiology 2004; 33: 743-748 | Retrospective, not prospective cohort. |
| Northern Manhattan Prospective Cohort Study | Stroke 2002; 33 (5): 1420 – 1423. | The study was designed as a case control study. |
| Oxford Vegetarian Study | Am J Clinical Nutrition 1999; 70: 525s-31s | Recruited volunteers so investigators could not influence sample. Focus on cardiovascular disease and cancer outcomes. |
| Study of German Vegetarians | Nutrition and Cancer 1988:11: 117-126 | Recruited volunteers so investigators could not influence sample. Not focused on cardiovascular disease outcomes. |
| Swedish Annual Level of Living Study | Am J Epidemiology 2004; 159: 655-662 | Core aim of the original survey was not to study cardiovascular outcomes. |
| UK Prospective Diabetes Study | Diabetes care 1998; 21:1271-1277 | Study is of people with diabetes |
| UK Vegetarians Study | BMJ 1996; 313: 775-779 | Study of volunteers recruited through several channels with investigators having little influence on the sample. Not focused on cardiovascular disease |
